# Altered Local Spontaneous Brain Activity in Juvenile Myoclonic Epilepsy: A Preliminary Resting-State fMRI Study

**DOI:** 10.1155/2016/3547203

**Published:** 2015-12-28

**Authors:** Sisi Jiang, Cheng Luo, Zhixuan Liu, Changyue Hou, Pu Wang, Li Dong, Chengqing Zhong, Yongxiu Lai, Yang Xia, Dezhong Yao

**Affiliations:** ^1^Key Laboratory for Neuroinformation of Ministry of Education, Center for Information in Medicine, High-Field Magnetic Resonance Brain Imaging Key Laboratory of Sichuan Province, University of Electronic Science and Technology of China, Chengdu 610054, China; ^2^Department of Neurology, Chongzhou People's Hospital, Chengdu 610000, China

## Abstract

*Purpose*. The purpose of this study was to evaluate the regional synchronization of brain in patients with juvenile myoclonic epilepsy (JME).* Methods*. Resting-state fMRI data were acquired from twenty-one patients with JME and twenty-two healthy subjects. Regional homogeneity (ReHo) was used to analyze the spontaneous activity in whole brain. Two-sample *t*-test was performed to detect the ReHo difference between two groups. Correlations between the ReHo values and features of seizures were calculated further.* Key Findings*. Compared with healthy controls, patients showed significantly increased ReHo in bilateral thalami and motor-related cortex regions and a substantial reduction of ReHo in cerebellum and occipitoparietal lobe. In addition, greater ReHo value in the left paracentral lobule was linked to the older age of onset in patients.* Significance*. These findings implicated the abnormality of thalamomotor cortical network in JME which were associated with the genesis and propagation of epileptiform activity. Moreover, our study supported that the local brain spontaneous activity is a potential tool to investigate the epileptic activity and provided important insights into understanding the pathophysiological mechanisms of JME.

## 1. Introduction

Idiopathic generalized epilepsies (IGE) are a group epilepsy syndrome clinically characterized by generalized tonic-clonic, myoclonic, and absence seizures [1]. Juvenile myoclonic epilepsy (JME) is a common subtype of IGE with an age-related onset of seizures, characterized by myoclonic jerks, tonic-clonic seizures, and less frequently absence seizures [2]. Standard interictal electroencephalography (EEG) features of JME consist of 4–6 Hz generalized spike-wave or polyspike-wave discharges (GSWDs) with a frontocentral predominance [2]. The corticothalamus circuit was considered a contributing factor in the propagation of GSWDs. Recently, simultaneous EEG-fMRI has been broadly used in epilepsy research, which demonstrated the metabolic alteration related to the GSWDs at the thalamus and wide cortex [3, 4]. Also, the myoclonic jerks, as a character of JME, may be caused by the motor circuitry hyperexcitability in EEG and fMRI studies [5, 6]. Thus, the thalamus and motor-related cortex may play an important role in JME. Resting-state fMRI (rs-fMRI) is extensively used to reflect spontaneous neuronal synchronization and intrinsic neurophysiologic process of the brain [7]. Many valuable findings of rs-fMRI were observed in neuropsychological diseases such as epilepsy [8–10], schizophrenia [11], and Alzheimer's disease [12].

As a data-driven method, regional homogeneity (ReHo) is able to measure the synchronization of activity in different brain regions by calculating the similarity of the time series of a given voxel with those of its nearest neighbors in a voxel-wise way [13]. This method has been applied to various clinical populations to investigate the functional modulations in the resting state [14–17]. Growing evidence have indicated increased synchronization in the epileptogenic zones during seizures and interictal state [18], which is believed to be involved in the generation of interictal activity [19]. In 2013, Zeng et al. observed that the significantly increased ReHo in mesial temporal lobe epilepsy might be related to the seizure genesis [20]. It is also interesting to note that, in generalized epilepsy with absence seizures [21] and generalized tonic clonic seizures [22], the ReHo features implicated the abnormality in striatothalamocortical network and default mode network. We wish to state here that, to best of our knowledge, there is not study focused on the local spontaneous activity according to the ReHo in JME. We, therefore, hypothesized that the ReHo characters would be altered between patients with JME and healthy controls, and the altered spontaneous brain activity may involve motor-related regions to response the myoclonic jerks in JME.

To evaluate the alterations of spontaneous brain activity in patients with JME, we investigated the ReHo features in a group of patients and healthy controls using rs-fMRI. We further assessed the influence of the ReHo alterations on the clinical factors in JME.

## 2. Materials and Methods

### 2.1. Subjects

Twenty-one (21) patients (mean age: 22.3 ± 5.7 years; mean years of duration: 10.9 ± 5.4; 14 females) with JME were recruited in Center for Information in Medicine, University of Electronic Science and Technology of China. All patients were diagnosed as JME based on the clinical and seizure semiology information consistent with the International League Against Epilepsy (ILAE) guidelines [23] by epileptologists (P. Wang and C. Zhong). All the routine brain neuroimaging including CT and MRI scanning showed no structural abnormalities; and scalp EEG demonstrated 4–6 Hz generalized spike-wave or polyspike-wave discharges. Twenty-two healthy controls were recruited as sex- and age-matched control group (mean age: 23.1 ± 8.7 years; 15 female). All the controls were free of neurological or psychiatric disorders. This study was approved by the ethical committee of the University of Science and Technology of China according to the standards of the Declaration of Helsinki. Written informed consent was obtained from each subject.

### 2.2. Data Acquisition

All subjects underwent MRI scanning in 3 T GE scanner with an eight-channel-phased array head coil (MR750; GE Discovery, Milwaukee, WI) in the MRI research center of University of Electronic Science and Technology of China. The resting-state functional data were collected using an echo-planar imaging sequence with the following parameters: repetition time (TR) = 2000 ms, echo time (TE) = 30 ms, flip angle (FA) = 90, field of view (FOV) = 24 × 24 cm^2^, matric = 64 × 64, and slice thickness = 4 mm with 0.4 mm gap and 255 volumes in each run. Axial anatomical T1-weighted images were acquired using a 3-dimensional fast spoiled gradient echo (T1-3D FSPGR) sequence (TR = 6.008 ms, TE = 1.984 ms, FA = 90, matrix = 256 × 256, FOV = 25.6 × 25.6 cm^2^, and slice thickness = 1 mm (no gap)) to generate 152 slices. During the examination, all subjects were instructed to be “relaxed, eyes closed” and kept awake and not to think of anything in particular.

### 2.3. Data Preprocessing

Preprocessing of fMRI dataset was conducted using the SPM8 software package (statistical parametric mapping available at http://www.fil.ion.ucl.ac.uk/spm). The first five volumes of each run was discarded to ensure magnetic field stabilization. The remaining 250 volumes were slice-time corrected and realigned. Any subject whose head motion exceeded 1.5 mm or/and 1.5 degrees was excluded in the following steps. We also assessed translation and rotation in both groups using the following formula [24, 25]: head motion/rotation = $(1/(M-1))\sum_{i=2}^{M}\sqrt{{\left|\Delta d_{x_{i}}\right|}^{2}+{\left|\Delta d_{y_{i}}\right|}^{2}+{\left|\Delta d_{z_{i}}\right|}^{2}}$, where *M* is the length of the time courses (*M* = 250 in this study); *x*_*i*_, *y*_*i*_, and *z*_*i*_ are translations/rotations at the *i*th time point in the *x*, *y*, and *z* directions, respectively, and Δ*d*_*x*_*i*__ = *x*_*i*_ − *x*_*i*−1_, and similar formula for *y*_*i*_ and *z*_*i*_. The realigned images were spatially normalized to the Montreal Neurological Institute (MNI) template using a 12-parameter affine transformation and resliced with voxel size of 3 mm × 3 mm × 3 mm. Then the images were smoothed with Gaussian kernel (8 mm full width at half maximum, FWHM). No temporal filtering was performed in this processing in consideration of the following analyses in full frequency band.

### 2.4. ReHo Analysis

The value of ReHo, which reflected the Kendall's coefficient of concordance of the Blood Oxygenation Level Dependent (BOLD) singles in local regions, was calculated using REST software (http://www.restfmri.net/forum/REST). We measured the homogeneity of time series of a center voxel in the cluster consisted of 27 nearest neighboring voxels. Then the resulted ReHo map was normalized by the mean ReHo value within the brain mask. Lastly, the map was smoothed with an isotropic Gaussian kernel (6 mm full width at half maximum).

### 2.5. Statistical Analysis

First, one-sample *t*-test was used in the ReHo value to evaluate the local spontaneous brain activity in both groups. Then, two-sample *t*-test was utilized to obtain the difference between JME and control group. The significance was set at *P* < 0.05 with FDR correction for multicompares and the cluster correction with a minimum cluster size of 23 voxels. Furthermore, the partial correlation analysis was performed to detect the relation between the value of ReHo of brain regions with significant difference and the clinical features including onset age and duration of epilepsy, with controlling effects of gender and age.

## 3. Results

Two of the recruited 21 patients were excluded from the ReHo analysis because of excessive head motion. There were no significant differences between the two groups in head motion and rotation (two-sample two-tailed *t*-test; *T* = 1.50, *P* = 0.14 for translational motion; and *T* = 1.15, *P* = 0.25 for rotational motion). The detailed demographic data of patients is shown in the Table 1.

### 3.1. Within-Group and Between-Group ReHo Values

**Figure 1:**
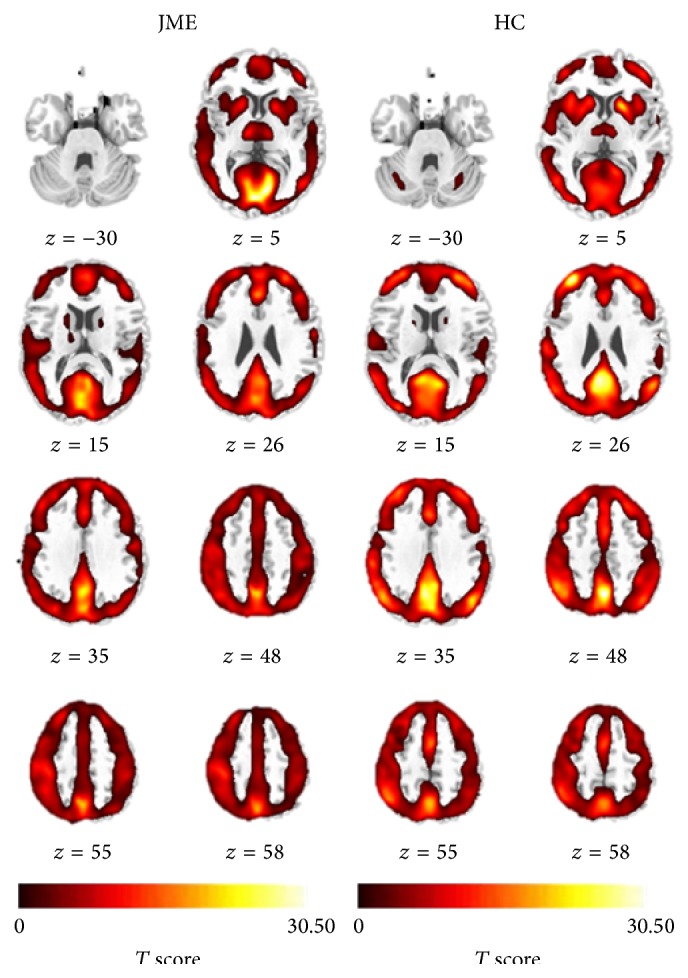
The ReHo results for patients with JME and healthy controls. The *T* values of one-sample *t*-test were showed in each group, respectively. HC: healthy control group.

The results of one-sample *t*-test in both groups was shown in Figure 1, respectively. The voxels with high ReHo value was found in bilateral distributed brain regions, including the major regions of the DMN, such as the medial frontal lobe, posterior cingulate cortex, and other regions including the thalami, putamen, caudate, insula, visual cortex, and cerebellum.

**Figure 2:**
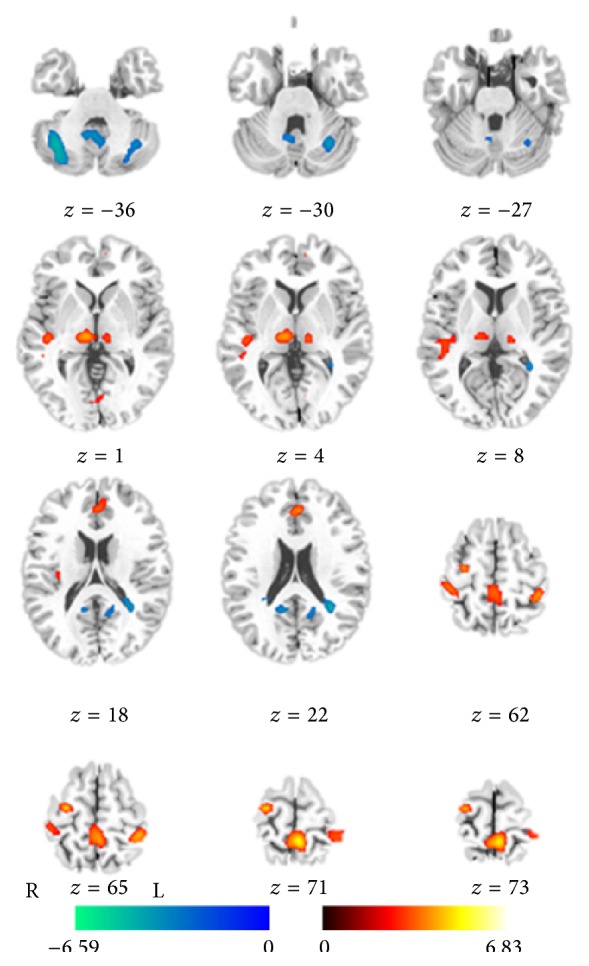
Statistic t-map showing the difference between the JME group and healthy control (two-sample *t*-test, *P* < 0.05 with FDR correction). The *T* scores were showed with hot color for positive values (JME > healthy controls) and cool colors for negative values (JME < healthy controls).

The significant differences of ReHo between two groups (*P* < 0.05, FDR corrected) were demonstrated in the Figure 2; and detailed information of clusters with difference was recorded in Table 2. Compared with the healthy controls, JME patients showed significantly increased ReHo values at left paracentral lobule, right precentral gyrus, bilateral postcentral gyrus, left anterior cingulate gyrus, right posterior insular/superior temporal gyrus, and bilateral thalami. Significantly decreased ReHo values were also shown in bilateral cerebellum, right cuneus, right precuneus, left middle frontal gyrus, and left superior marginal gyrus.

### 3.2. Correlation Analyses

**Figure 3:**
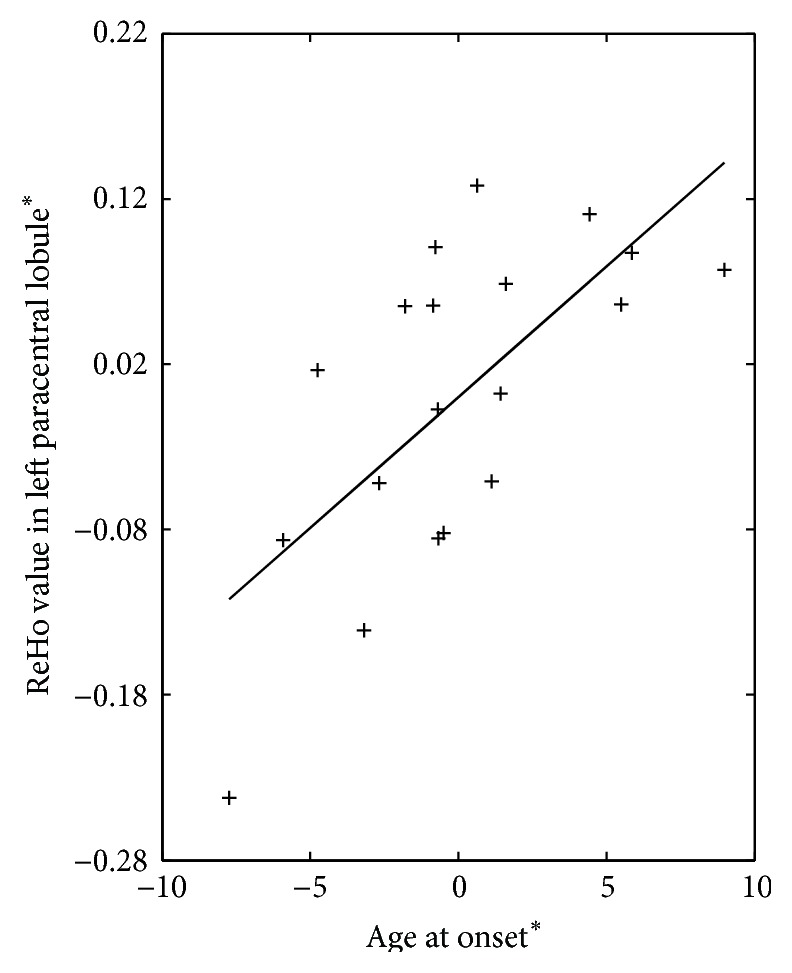
The relation between ReHo value in left paracentral lobule and age at onset in patients with JME. +: the coordinate value implicates the residuals after controlling for the influence of the gender and age (linear regression with covariates including gender and age).

The relation analysis of mean ReHo in JME patients and clinical features revealed a significant positive correlation between age of onset and ReHo value in left paracentral lobule (*r* = 0.68, *P* = 0.002) (Figure 3). No significant correlations were observed between duration of disease and ReHo value in all regions with difference between groups.

## 4. Discussion

The current study originally investigated the alterations of regional synchronization in patients suffering from JME compared with a group of age and gender-matched controls using the resting-state fMRI. We performed a voxel-based comparison of ReHo value to identify the alterations in local spontaneous brain activity. Compared to healthy controls, patients with JME showed a significant increased ReHo value in bilateral thalami and some cortex regions related to motor function, including primary motor and sensory cortex, insula, and anterior cingulate cortex; the significant reduction of ReHo was also observed in cerebellum and occipitoparietal lobe. Furthermore, our study showed that the high ReHo value in the left paracentral lobule was linked to the older age of onset in patients. ReHo represents the temporal similarity of the signals in a given local region, which may reflect spontaneous brain activity [13]. The epileptogenic zones might be related to the abnormal synchronization of neural electrical activity [26]. Consistent with the previous researches, which suggested abnormalities in frontal lobe and thalamus in JME [27], our investigation suggests the abnormality of thalamomotor cortical network in JME which were associated with the genesis and propagation of epileptiform activity. We, hence, provided important insights into understanding the pathophysiological mechanisms of JME.

The regions related to motor function and bilateral thalami had increased ReHo values in JME patients compared to controls in this study. In fact, more and more structural and functional evidences supported that the thalami and motor regions play important role in JME [27–29]. Thalami in functional level are proposed to be involved in the propagation of generalized epileptic discharges in JME [27]. It is important to state that recent studies of simultaneous EEG and fMRI have consistently shown altered metabolic activity at cortical and subcortical regions, especially the thalami, in generalized epilepsy [3]. A study of MRS showed an absolute reduction of N-acetyl aspartate (NAA) value in the thalamus in JME [30], which may reflect the abnormality of metabolite at the thalami. This observation may result from a reflection of neuronal marker by NAA in the injured neuron [31]. In addition to the functional abnormalities in JME patients, structural changes were revealed in many studies. The structural researches centered on voxel-based morphometry demonstrated reductions in gray matter volume of the thalami [32] and a negative correlation between thalamic gray matter volume and the duration of epilepsy [33]. Diffused tensor imaging studies of patients with JME particularly in thalamocotical fiber white matter indicated a significant decreased diffusion feature and fractional anisotropy [34]. Recently, MRI studies in JME illustrated structural and functional abnormality in the motor cortex in patients with JME [5, 35]; in particular, the functional changes were observed in the unaffected siblings of patients, suggesting the underlying genetic risk of JME [35]. In addition, ReHo was identified with a neuroimaging marker to investigate the neural activity [36]. The increased ReHo value was responsible for seizure genesis and propagation, such as increased ReHo in the ipsilateral mesial temporal structure in patients with mesial temporal lobe epilepsy [37] and in the central, premotor, and prefrontal regions in patients with benign epilepsy with centrotemporal region spikes [38]. Together with our findings of increased ReHo in thalami and motor-related cortex, we inferred that these findings may reflect dysfunction of the thalamocortical network associated with epileptic activity in JME.

Furthermore, patients with JME also exhibited a stronger magnitude of the regional spontaneous activity in left paracentral lobule, and was positively correlated with the age of onset. These findings would reflect the influence of seizures on neural plasticity in JME patients. We presumed that patients with earlier age of seizures have more compensatory mechanisms of maturation, as the younger brain is more plastic and can accommodate more easily than an adult mature brain [39]. In addition, recent research observed that ReHo across the entire brain was higher in children than in adolescents and adults in healthy controls [40]. Thus, based on the enhanced influence of myoclonic seizures on the ReHo magnitude at left paracentral lobule, the positive relation might indicate that patients with the later onset age, who illustrated higher ReHo value, have low effect resulting from development.

Meanwhile, the alterations of local regional synchronization of brain activity may provide a possible measurement to assess the potential epileptogenic region. The increased ReHo in ipsilateral parahippocampal gyrus was found in the mesial temporal lobe epilepsy, suggesting an important implication for the seizure genesis and propagation [20]. Functional abnormality at motor cortex is considered to play a critical role for jerks during myoclonic seizures. Thus, the increased ReHo at the primary motor and sensory cortex may imply that the local brain spontaneous activity contributed to localize the epileptogenic zone in JME.

Another remarkable finding in our study is the decreased ReHo in cerebellum and occipitoparietal lobe. In fact, there are more and more studies showing that cerebellum may be important for cognitive beyond classical involvement of motor coordination [41]. In addition, the cerebellum plays a role in multiple functional domains: cognitive, affective, and sensory function [42, 43]. It was also observed that the altered ReHo value was present in patients with absence seizures and generalized tonic clonic seizures. In line with these studies, our finding of decreased ReHo in the cerebellum in JME patients may hint the potential spontaneous neural activity in cerebellum related partially to the neurological JME. Also, decreased ReHo was observed in the occipitoparietal lob, especially at right precuneus lobe. As acknowledged by us, these regions are the salient posterior nodes of default mode network (DMN), which maintains the baseline of spontaneous neuronal activities related to self-awareness, episodic memory, and interactive modulation between internal mental activities and external tasks [44]. The altered spontaneous BOLD fluctuation in DMN has been observed in patients with epilepsy [22, 45, 46]. In this study, we noted that the decreased ReHo in DMN may be associated with the functional impairments in cognitive processes in patients with JME. However, further study and analysis of DMN would be considered in the future.

Notwithstanding the results of this study, there are several limitations inherent in our research. One limitation concerns the sample size which is relatively small. A second limitation is in regard to the possibility of interictal epileptic discharges influencing the resting-state functional connectivity in patients with epilepsy. The EEG, however, was not recorded during fMRI scans, so it is impossible to rule out the effect of confounding epileptic discharges. Therefore, simultaneous EEG-fMRI would be considered in the further study to clarify the relationship between ReHo and the interictal epileptic discharges. Third, the antiepileptic drugs taken by some patients may confound the finding in this study. In general, the antiepileptic drugs may directly act on the neurotransmitters in brain, so the spontaneous activity might be influenced directly by antiepileptic drugs. In addition, the cognitive impairment of antiepileptic drugs have been found in many studies; the abnormal cognition related to the antiepileptic drugs might contribute to the altered spontaneous brain activity in epilepsy.

## 5. Conclusion

In this research, we studied the patter of regional hemodynamic synchronization in the JME patients by using ReHo analysis in resting-state fMRI. Patients with JME demonstrated altered regional synchronization in bilateral thalami, motor-related cortex, the posterior DMN regions, and cerebellum. In addition, the enhancing magnitude of the regional spontaneous activity with increasing age of onset was observed in the left paracentral lobule in patients. The increased ReHo composed in the thalamomotor cortical network might indicate that the local brain spontaneous activity contributed to localize the epileptogenic zone in JME. These results, which would support ReHo methods as a potential tool to detect intrinsic epileptic activity, provide important insights into understanding the pathophysiological mechanisms of JME.

## Figures and Tables

**Table 1:** Demographic data of 19 juvenile myoclonic epilepsy patients.

Number	Gender	Age (year)	Age at seizure onset (year)	Frequency of GSWDs (Hz)	History/family history	Antiepileptic drugs
1	F	17	13	2	Sister with JME	VPA
2	F	17	3	3~3.5	Sister with JME	VPA
3	M	26	8	6	—	MVP
4	F	20	6	2	—	VPM, LTG
5	F	27	16	4	Daughter with GTCS	VPA
6	F	16	13	3	—	LTG
7	F	22	14	3	—	VPA
8	F	23	7	5	—	VPA
9	M	15	5	3~3.5	—	—
10	F	17	10	4~4.5	—	VPA
11	F	29	10	3~3.5	—	VPM
12	F	33	20	6.5~7	—	VPM, LTG
13	M	18	14	5	—	VPM
14	M	22	8	4	—	VPM
15	F	21	11	2	—	VPM
16	F	19	12	2	—	MVP
17	M	34	14	3	—	VPM, TCD
18	F	25	21	3~3.5	Uncle with GTCS	VPM
19	M	30	9	3.5~4	—	TOP, CBZ

M: male; F: female; VPA: valproic acid; VPM: valpromide; MVP: magnesium valproate; LTG: lamotrigine; TOP: topiramate; CBZ: carbamazepine.

**Table 2:** Brain regions showing abnormal regional homogeneity in patients with JME.

Brain region	MNI coordinates (*x*, *y*, *z*)	BA	*T* value	Voxel number
*Patients > controls*				
Paracentral lobule L	−3, −45, 69	5	6.08	151
Precentral gyrus R	27, −15, 66	6	5.61	45
Thalamus R	9, −21,0	—	5.15	48
Thalamus L	−8, −23,1	—	4.98	38
Postcentral gyrus L	−39, −39,63	2	4.38	48
Anterior cingulate gyrus L	−3, 36, 24	32	4.69	34
Posterior insular/superior temporal lobule R	45, −33, 9	41	4.03	32
*Patients < controls*				
Cerebellum_Crus2 R	33, −75, −42	—	6.57	298
Middle occipital L	−36, −66,30	19/39	4.71	228
Cuneus R	15, −66,36	7	5.16	190
Cerebellum_Crus1 L	−30, −63, −33	—	4.82	208
Calcarine L	−12, −57,18	23/30	4.40	47
Supramarginal R	66, −24,27	2/48	4.03	54

MNI: Montreal Neurological Institute; BA: Brodmann area; L: left; R: right.
